# Distinct pattern of microsusceptibility changes on brain magnetic resonance imaging (MRI) in critically ill patients on mechanical ventilation/oxygenation

**DOI:** 10.1007/s00234-021-02663-5

**Published:** 2021-03-01

**Authors:** Majda M. Thurnher, Jasmina Boban, Martin Röggla, Thomas Staudinger

**Affiliations:** 1 Department for Biomedical Imaging and Image-Guided Therapy, Medical University of Vienna, Waehringer Guertel 18-20, A-1090 Vienna, Austria; 2 Faculty of Medicine, University of Novi Sad, Hajduk Veljkova 3, Novi Sad, SR-21000 Serbia; 3 Department of Emergency Medicine, Medical University of Vienna, Waehringer Guertel 18-20, A-1090 Vienna, Austria; 4 Department of Internal Medicine I, Medical University of Vienna, Waehringer Guertel 18-20, A-1090 Vienna, Austria

**Keywords:** Microbleeds, Brain, Magnetic resonance imaging (MRI), Mechanical ventilation, Extracorporeal membrane oxygenation (ECMO)

## Abstract

**Purpose:**

Over the years, interesting SWI abnormalities in patients from intensive care units (ICU) were observed, not attributable to a specific cause and with uncertain clinical significance. Recently, multiple SWI-hypointense foci were mentioned related to neurological complications of SARS-COV-2 infection. The purpose of the study was to describe the patterns of susceptibility brain changes in critically-ill patients who underwent mechanical ventilation and/or extracorporeal membrane oxygenation (ECMO).

**Methods:**

An institutional board-approved, retrospective study was conducted on 250 ICU patients in whom brain MRI was performed between January 2011 and May 2020. Out of 48 patients who underwent mechanical ventilation/ECMO, in fifteen patients (median age 47.7 years), the presence of SWI abnormalities was observed and described.

**Results:**

Microsusceptibilities were located in white-gray matter interface, in subcortical white matter (U-fibers), and surrounding subcortical nuclei in 13/14 (92,8%) patients. In 8/14 (57,1%) patients, SWI foci were seen infratentorially. The corpus callosum was affected in ten (71,4%), internal capsule in five (35,7%), and midbrain/pons in six (42,8%) patients.

**Conclusion:**

We showed distinct patterns of diffuse brain SWI susceptibilities in critically-ill patients who underwent mechanical ventilation/ECMO. The etiology of these foci remains uncertain, but the association with mechanical ventilation, prolonged respiratory failure, and hypoxemia seems probable explanations.

## Introduction

MRI has been proven to be the method of choice for the detection of neurological complications in critically ill patients. Susceptibility-weighted imaging (SWI) is a recently introduced magnetic resonance imaging (MRI) technique that provides improved detection of substances that cause susceptibility effects, such as iron, blood products, calcification, and air [1]. Studies have shown the superiority of SWI when compared with T2* for the detection of microsusceptibility changes in the brain, such as microhemorrhages in microangiopathy, moyamoya syndrome, angiitis, diffuse axonal injury, anticoagulant therapy, cerebral amyloid angiopathy, Fabry disease, embolism, brain radiation, and posterior reversible encephalopathy syndrome (PRES) [2, 3]. Cerebral microbleeds (CMBs) are small-sized (less than 10 mm in diameter), focal, perivascular, hemosiderin depositions that are detected as round or ovoid, SWI-hypodense foci within the cerebral parenchyma. In cerebral amyloid angiopathy (CAA), microbleeds are predominantly located in the subcortical white matter but sparing the basal ganglia and pons [4]. CMBs associated with hypertonia will mostly be seen in the basal ganglia and pons. Recently, multiple SWI-hypointense foci have also been mentioned in case reports related to the neurological complication of SARS-COV-2 infection [5].

Over the years, we have observed interesting SWI abnormalities in patients referred from intensive care units, which could not be attributed to a specific cause and had uncertain clinical significance. After a search of the hospital database, several patients with identical SWI findings were identified, interestingly, all with a history of mechanical ventilation and/or ECMO.

Neurological complications of mechanical ventilation and ECMO have been described mostly in anesthesiologic literature, including ischemic changes in the subcortical gray matter and deep brain structures, subarachnoid hemorrhages, watershed infarctions, and diffuse petechial hemorrhages [6–9]. Intracerebral bleeding is a feared complication of ECMO therapy. Its incidence has, however, decreased over the past 20 years from nearly 20% [11] to 2–7%, likely due to improved anticoagulation management, as well as to technical progress [10, 11].

In a retrospective manner, the aim of this study was to (a) describe the extent and location of SWI susceptibility changes in the brain of patients who underwent mechanical ventilation and/or ECMO therapy and (b) identify the risk factors and clinical significance of the imaging findings (with respect to the outcome and neurological sequalae).

## Methods

### Subject selection

Patients included in the study were identified from the institutional radiology database. Between January 2011 and May 2020, a total of 250 patients referred from intensive care units underwent MRI of the brain. Patients without SWI in the MR protocol were excluded (29 patients). MR exams in 221 patients were analyzed, and 166 patients with no record of mechanical ventilation were excluded. Seven patients were further excluded: angiocentric lymphoma (1 patient), DIC and hemophagocytic syndrome (1 patient), small hemorrhagic fungal abscesses (1 patient), microbleeds after hearth transplantation (1 patient), high blood pressure with known microbleeds (1 patient), and low-quality images (2 patients).

SWI of 48 patients who underwent mechanical ventilation or ECMO was analyzed for the presence of microsusceptibilities. SWI microsusceptibilities were identified in 14 patients. The patient selection process is shown in Table 1.

**Fig 1:**
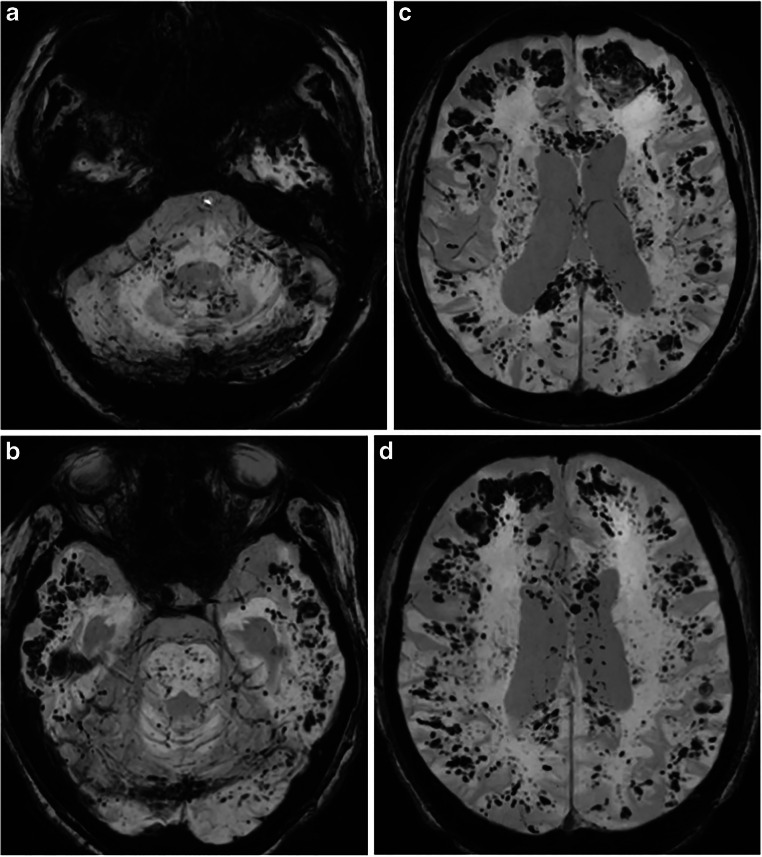
Susceptibility-weighted MR images (MRI) of the brain in a 55-year-old female patient with ARDS after abdominal surgery, demonstrate diffuse susceptibilities (type I) in the cerebellum (**a**), pons (**b**), gray and white matter interface (**b**,**c**,**d**), corpus callosum (**b**)

Patient information data, including demographics (age, gender, and underlying diseases), indication for mechanical ventilation, type of mechanical ventilation, laboratory and clinical findings, outcome, imaging data, and clinical follow-up data, were collected from the electronic medical record and hospital database (Table 2). Indications for mechanical ventilation were classified as respiratory (due to respiratory failure with refractory hypoxia and/or hypercapnia) or cardiac (due to cardiovascular failure as a result of myocarditis, hypotension, congenital heart disease, etc.). This retrospective, descriptive study was approved by the institutional review board.

**Table 1:**
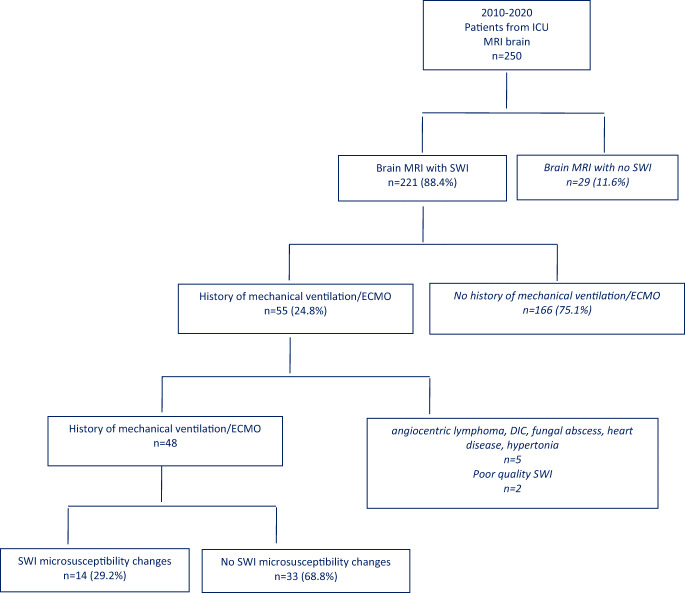
The flowchart demonstrates the steps of the search for patients included in the study

**Table 2:** Characteristics of critically-ill patients who underwent mechanical ventilation/ECMO and MRI study

No.	Age	Gender	Underlying disease	SWI pattern type
1	54	F	Pneumococcal meningitis, ARDS	Type II-subcortical only
2	22	M	Cystic fibrosis, lung transplantation	Type I-diffuse
3	20	F	Drug abuse, ARDS	Type I-diffuse
4	55	F	Diverticulitis, abdominal surgery, ARDS	Type I-diffuse
5	57	M	Liver transplant	Type II-subcortical only
6	69	M	Liver transplant	Type I-diffuse
7	27	F	H1N1 pneumonia/ARDS	Type I-diffuse
8	45	F	Sarcoidosis, lung transplant, CMV pneumonia, ARDS	Type III-diffuse, predominantly corpus callosum
9	55	M	AML, BMT, septic shock	Type I-diffuse
10	49	F	Pneumonia/ARDS	Type III-corpus callosum, basal ganglia
11	23	M	Heart surgery/ARDS	Type II-subcortical, pons
12	46	M	Psychiatric disease	Type III-corpus callosum only
13	47	M	Influenza A, ARDS	Type III-corpus callosum
14	45	F	COVID-19, ARDS	Type I-diffuse

### Imaging protocol

Imaging studies were performed during the period from January 2010 to May 2020, on 1.5T and 3T MR scanners (Siemens Trio Tim, Erlangen, Germany and Philips Ingenia, Philips Healthcare, Netherlands). Twenty-two MR exams were performed in 14 patients. Fourteen MR exams were performed on a 1.5 T scanner, and eight exams on a 3 T scanner. Eight patients had one exam only, 3 patients had two exams, and 3 patients had three exams.

Only in one patient exams were performed on different scanners.

In all patients, standard axial fluid attenuation inversion recovery (FLAIR), 3D T1-weighted (T1W), coronal T2-weighted (T2W), diffusion-weighted imaging (DWI), and susceptibility-weighted imaging (SWI) were performed. The parameters for the SWI sequence on the 3T MR unit (Trio Tim, Siemens) were field of view (FOV) 220×172×130mm (APxRLxHF); voxel size (VS) 0.6×0.6×1.5mm; reconstruction matrix (RM) 960; time of echo (TE)/ repetition time (TR) 7.12ms/31ms; and flip angle (FA) 17°, with no water or fat suppression, slice thickness 1.5mm.

The parameters of the SWI sequence on the 1.5T MR unit (Philips Ingenia, The Netherlands) were FOV 230×185×150mm; voxel size 0.85×1.01×2mm; TE/TR 12/shortest; and FA 20°, without water or fat suppression, slice thickness 2mm.

### Imaging analysis

A total of 22 MR examinations in 14 patients were analyzed on a PACS workstation by two experienced neuroradiologists (MMT, JB). Interpretations were determined in consensus.

The following imaging characteristics on SWI were recorded: (a) presence of SWI abnormalities, (b) size of SWI abnormalities, and (c) location of SWI abnormalities. The location of the abnormalities was divided into supratentorial white matter, corpus callosum, pons/midbrain, cerebellum, basal ganglia, and thalamus. Findings on conventional sequences and diffusion-weighted imaging (DWI) were also recorded.

## Results

A total of 14 adult patients, seven women and seven men, mean age, 47.7 years (range 20-69 years), with brain microsusceptibility changes were identified. Respiratory failure was the indication for mechanical ventilation/ECMO in ten patients (71,4%), while cardiovascular failure was the indication in four patients (28,6%). Respiratory failure was caused by cystic fibrosis in two patients, influenza in one patient, sarcoidosis in one patient, pneumonia in five patients, and SARS-CoV-2 infection in one patient. Cardiovascular failure was caused by hemorrhagic shock in one patient, heart failure in one, and congestive cardiomyopathy in two patients. Seven patients underwent ECMO treatment for a median of 16.2 days (range, 3-35 days). The clinical data of the patients are summarized in Table 2.

### MRI findings

In all 14 patients, an identical pattern of innumerable SWI hypointense foci was identified. These were located in the white and gray matter interface, both in the subcortical white matter (U-fibers) and surrounding subcortical nuclei in 13 of 14 (92,8%) patients. In eight of 14 (57,1%) patients, SWI foci were seen infratentorially in the cerebellar hemispheres. The corpus callosum was affected in ten (71,4%), the internal capsule in five (35,7%), and the midbrain/pons in six (42,8%) patients. Four patients had a previous MR (performed before the ICU stay) exam with no SWI abnormalities. SWI hypointense foci were not detectable on standard sequences (FLAIR, T2WI, and T1WI). None of the SWI foci demonstrated restricted diffusion. In six (42,8%) patients who had more than one MR exam, there was no change in the number, distribution, or size of the SWI hypointense foci.

Three patients had an intracerebral hematoma, one patient had hypoxic-ischemic brain injury, two patients had imaging findings related to posterior reversible encephalopathy syndrome (PRES), and, in two patients, infarcts were present.

**Fig. 2:**
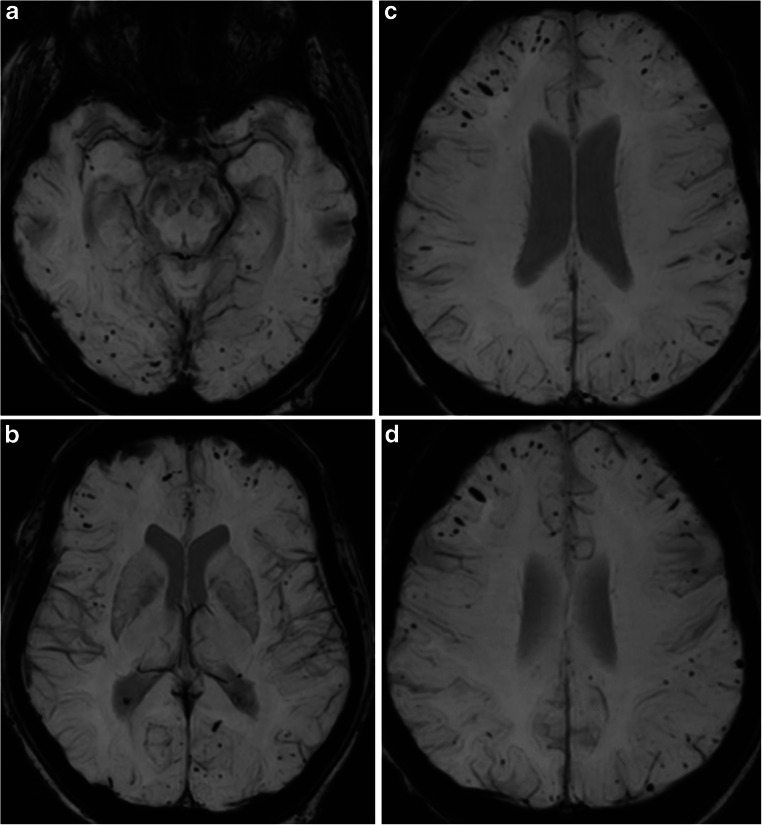
In a 54-year-old female patient with pneumococcus meningitis and ARDS, who underwent mechanical ventilation with ECMO, SWI (**a**-**d**) show bilateral susceptibilities in the subcortical white matter (type II). MR exam performed 1 year earlier showed no SWI abnormalities (not shown)

**Fig. 3:**
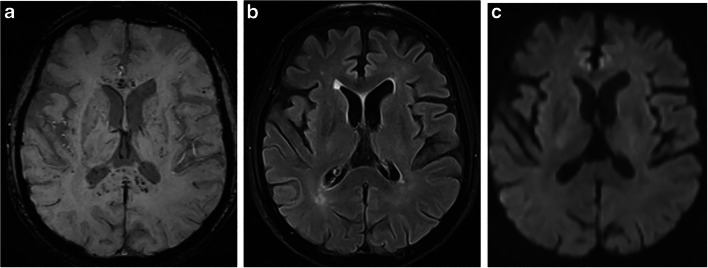
In a 54-year-old male liver transplant patient with respiratory failure and mechanical ventilation, SWI (**a**) shows numerous microsusceptibilities in the corpus callosum (type III). Axial fluid-attenuated inversion-recovery (FLAIR) and diffusion-weighted (DWI) images (**c**) do not show any abnormality

Based on the extent and location of SWI foci, three patterns were identified: (1) pattern I, diffuse pattern, with hypointense foci on the gray and white matter interface, in the midbrain and cerebellar hemispheres, as well as in the corpus callosum (observed in 7 of 14 patients) (Fig. 1); (2) pattern II, subcortical pattern, with hypointense foci in the white and gray matter interface, both in the subcortical white matter (U-fibers) and surrounding subcortical nuclei (observed in 3 of 14 patients), no SWI abnormalities infratentorial and corpus callosum (Fig. 2); and (3) pattern III, corpus callosum pattern, with SWI foci identified in the corpus callosum (observed in 3 of 14 patients) (Fig. 3). Two patients had corpus callosum involvement only, and one patient had lesions in the pons.

### Clinical outcome

Seven patients (50%) were discharged from the intensive care unit. No major neurological consequences were recorded in survivors. It must be considered that no neuropsychiatric evaluation for metabolic toxic encephalopathy and post ICU syndrome was performed, leading to depression and dementia-like cognitive impairment. Seven of 14 patients (50%) had a lethal outcome, six as a consequence of the underlying disease. One patient died due to a major intracerebral hematoma.

## Discussion

Advances in critical care have led to improved survival from acute illnesses that have historically been associated with high mortality, including respiratory failure. Invasive and non-invasive mechanical ventilation have become a well-established treatment option for patients with respiratory insufficiency following thoracic and cardiovascular surgery. Ventilatory support is usually provided as positive pressure ventilation via a non-invasive interface or an endotracheal tube, either as assisted or assist-control ventilation. Positive end-expiratory pressure (PEEP) is used to maintain or increase the end-expiratory lung volume (EELV) to minimize end-expiratory atelectasis, improve oxygenation, and impede ventilator-associated lung injury. CPAP, or continuous positive airway pressure, refers to spontaneous ventilation with a positive airway pressure maintained throughout the whole respiratory cycle by the application of PEEP. In the case of severely impaired gas exchange refractory to mechanical ventilation, extracorporeal membrane oxygenation (ECMO) is increasingly used as a lifesaving therapeutic option [12, 13].

A strikingly identical pattern of diffuse, symmetrical, innumerable foci of SWI hypointensity was observed on the gray and white matter interface, the corpus callosum, the cerebellum, and the midbrain/pons, in 14 critically-ill patients who underwent mechanical ventilation in our study. The etiology of the SWI microsusceptibility foci in critically ill patients remains uncertain. Although the most commonly used term for these changes is “microbleeds,” the underlying pathophysiology and the exact etiology of neuronal and/or vascular injury are not yet fully understood. In the majority of reported patients, lesions are distributed supratentorially and diffusely and do not seem to be related to any specific vascular territory.

Several hypotheses have been discussed, ranging from hemorrhagic transformation of ischemic lesions, the result of thrombocytopenia, disseminated intravascular coagulation (DIC), small gaseous emboli, and platelet dysfunction. The largest study on critical illness-associated cerebral microbleeds included 12 patients with respiratory failure [14]. Interestingly, only three of 12 patients received extracorporeal life support. MRI showed extensive microbleeds, diffusely involving the juxtacortical white matter and the corpus callosum, but sparing the cortex, the deep and periventricular white matter, the basal ganglia, and the thalami. Histopathologic analysis of one patient revealed small clusters of free red blood cells and red blood cells within macrophages, consistent with recent microbleeds, as well as surrounding degradation of myelinated fibers. Notably, 11 of 12 patients in that particular study had coagulopathy.

Extracorporeal membrane oxygenation (ECMO) is a method of respiratory support in patients with respiratory or circulatory failure. Studies have reported an increased prevalence of neurologic injury in patients who receive ECMO, ranging from 11 to 50% [15–19]. The most commonly reported neurological complications during ECMO are intracerebral and/or subarachnoid hemorrhages and ischemic strokes. In a recently published systematic review and meta-analysis of brain injury and neurological outcome of patients undergoing extracorporeal cardiopulmonary resuscitation, neurological complication was reported in 23 studies (441 events) with a frequency of 27% [16].

In a study by Chow et al., two patients with H1N1 influenza and ECMO-related cerebral injury are described, one with a major intracerebral hematoma and the other with multiple foci of microsusceptibility on the gray and white matter interface [17]. Another case is a case of a 30-year-old woman with influenza-A pneumonia and ARDS who was on ECMO for 12 days and developed multiple microbleeds at the cortico-subcortical junction and deep white matter and one larger hemorrhage [18]. Microsusceptibility changes described could be explained by vasogenic edema and vasodilatation in cerebral capillaries due to increased venous pressure during ECMO ventilation.

Small gaseous emboli were proposed as a pathological substrate for the changes in one study [16]. At autopsy, multiple foci were associated with ischemic lesions and not hemorrhage, thus, raising the probability that these changes were the result of air trapped in the arterioles [16]. However, air emboli would gradually decrease in the size over time (hours to days). In our study, we also did not observe any change on the follow-up examinations.

The other proposed cause of microsusceptibility changes could be hemosiderin deposition in macrophages adjacent to the vascular walls. Microvascular thrombi in the microvessels of the brain can be lysed during the process of angiophagy. Endothelium adjacent to the site of the occlusion completely engulfs the embolus, the endothelial barrier opens, and the embolus extrudes into the surrounding parenchyma [20], 24 hours after the occlusion. Soon after the extrusion, the emboli are engulfed by pericytes and subsequently degraded by parenchymal microglia [21].

A recently described entity, high-altitude cerebral edema, was reported to have similar microsusceptibility changes in the splenium of the corpus callosum and white matter, both supra- and infratentorially [21, 22]. The major factor in the pathophysiology of this disorder is vasogenic edema due to blood-brain barrier disruption, which can also be induced by hypoxemia [21]. Deep hypoxemia causes cerebral vasodilation with increased cerebral capillary hydrostatic pressure and promotes extracellular (vasogenic) edema and disruption of the blood-brain barrier. Vasogenic edema typically and preferentially spreads along the fiber tracts, so the predilection sites for this edema type are the corpus callosum and the white matter [23]. However, tissue hypoxia varies among individuals, so not every person is equally susceptible to hypoxia-induced changes [24]. In contrast to typical ICU patients with respiratory failure, high altitude exposure leads to hypocapnia, caused by the hyperventilatory response to high altitude [25]. Nevertheless, as argumented further below, rapid shifts in PCO2 and PaO2 may be a common feature of both conditions.

In patients with severe hypercapnia due to respiratory failure, rapid reductions in carbon dioxide levels have been implicated as a possible cause of neurologic injury due to the resultant vasoconstriction and acute reductions in cerebral blood flow [8, 11]. Rapid conversion from hypercapnia to normocapnic or hypocapnic states may also lead to impaired cerebral autoregulation, further increasing the risk of complications such as cerebral ischemic injury [8, 11]. In the last few years, the potential negative prognostic influence of hyperoxygenation or rapid overcorrection of hypoxia has gained a lot of focus [26]. Short phases of hyperoxygenation can be difficult to avoid after the emergency-institution of ECMO or even after intubation of a patient with severe respiratory failure. Up to now, no specific neuroanatomical pattern has been described for this condition. It is not known whether hyperoxygenation after severe hypoxia occurs regularly in the course of the rescue of a victim of high-altitude cerebral edema (HACE).

The complex interaction between mechanical ventilation and cerebral hemodynamics appears to be influenced by multiple patient-specific factors. In the normal brain, positive-pressure ventilation does not significantly alter intracranial pressure, cerebral oxygenation, or perfusion. In one study, the effect of PEEP on intracranial pressure (ICP) and cranial perfusion pressure (CPP) was analyzed in a large population of patients with acute brain injury and various categories of acute lung injury [27]. The study showed that, for every centimeter H2O increase in PEEP, there was a 0.31 mmHg increase in ICP and a 0.85 mmHg decrease in CPP [27]. The impact of PEEP on ICP is lessened if lung compliance is low. However, the “trade off” of lung protection (permitting some hypercapnia) and brain protection (avoiding abrupt hypercapnia, sometimes rapidly inducing hypocapnia) needs to be considered [28].

A recent multicenter study of 11,972 patients who received ECMO for respiratory failure showed that a high relative decrease in PaCO2 in the first 24 hours of ECMO initiation appears to be independently associated with an increased incidence of neurological complications, including seizures, stroke, intracranial hemorrhage, and brain death [29]. A large decrease in PaCO2 in the first 24 hours led to vasoconstriction and impaired cerebral perfusion. Brain hemorrhages were observed in 3.5% of patients. Imaging findings were not discussed in that particular study. Recently published autopsy study in patients who underwent ECMO demonstrated acute brain injury in 68% of patients with the most common type being hypoxic-ischemic brain injury, followed by intracranial hemorrhage (24%) and ischemic infarct (16%) [30]. No correlation was performed with imaging studies, and no information was given on microbleeds.

Central nervous system (CNS) involvement is a known feature of viruses with neurotropic characteristics. Since the COVID-19 outbreak, several reports on neurological manifestations of the diseases have been published [5, 31, 32]. In reports on critically-ill patients with COVID-19, leukoencephalopathy and SWI microhemorrhages have been mentioned in up to 30% of patients [5, 32–34]. In one of our critically-ill patients with COVID-19, the identical MRI pattern of multiple SWI foci was observed also (Table 1, patient #14 and #15). As the full clinical spectrum of COVID-19-associated neurological manifestations continues to be elucidated, caution is advised in interpreting diffuse SWI microhemorrhages in patients with severe acute respiratory syndrome coronavirus 2 (SARS-CoV-2) infection of the brain. The association with mechanical ventilation, prolonged respiratory failure, and hypoxemia is the only one explanation for the MR findings [34]. The other possible pathophysiology includes systemic endotheliitis and thrombotic microangiopathy [33].

Our study has several limitations; the retrospective nature of your work, the scarce number of patients, and the lack of clinical correlates (mechanical ventilation parameters, hemodynamic status, neurological conditions, etc.). This study did not focus on detailed laboratory and neurologic findings and their relation to imaging findings. The results however encouraged a larger prospective study in our institution.

## Conclusion

Larger prospective studies are necessary to delve deeper into the pathophysiology of brain SWI microsusceptibility changes in critically-ill patients with ventilation support and to reveal possible common clinical and laboratory characteristics. Given the frequency and severity of neurologic complications observed in COVID-19 patients in the ongoing pandemic, caution is needed to avoid misinterpretation of MR imaging findings in patients on mechanical ventilation.
